# Effects of honey supplementation on inflammatory markers among chronic smokers: a randomized controlled trial

**DOI:** 10.1186/s12906-017-1703-6

**Published:** 2017-03-28

**Authors:** Wan Syaheedah Wan Ghazali, Aminah Che Romli, Mahaneem Mohamed

**Affiliations:** Department of Physiology, School of Medical Sciences, Universiti Sains Malaysia, 16150 Kubang Kerian, Kelantan Malaysia

**Keywords:** Chronic smokers, Honey, Inflammatory markers, C-reactive protein, Tumor necrosis factor-α

## Abstract

**Background:**

Honey has been demonstrated to possess anti-inflammatory property. This is a randomized, controlled, open-label trial to determine the effects of 12-week honey oral supplementation on plasma inflammatory markers such as high sensitive C-reactive protein, interleukin-6 and tumor necrosis factor-α among chronic smokers.

**Methods/design:**

A total of 32 non-smokers and 64 chronic smokers from Quit Smoking Clinic and Health Campus, Universiti Sains Malaysia participated in the study. Smokers were then randomized into 2 groups: smokers with honey group that received Malaysian Tualang honey (20 g/day daily for 12 weeks) and smokers without honey group. Blood was obtained from non-smokers and smokers at pre-intervention, and from smokers at post-intervention for measurement of the inflammatory markers.

**Results:**

At pre-intervention, smokers had significantly higher high sensitive C-reactive protein than non-smokers. In smokers with honey group, tumor necrosis factor-α was significantly increased while high sensitive C-reactive protein was significantly reduced at post-intervention than at pre-intervention.

**Conclusion:**

This study suggests that honey supplementation has opposite effects on tumor necrosis factor-α and high sensitive C-reactive protein indicating the inconclusive effect of honey on inflammation among chronic smokers which needs further study on other inflammatory markers.

**Trial registration:**

The Trial has been registered in the Australian New Zealand Clinical Trials Registry: ACTRN12615001236583. Registered 11 November 2015 (Retrospectively Registered).

## Background

Cigarette smoking has been reported as one of the major causes of cardiovascular disease [1]. Elevated inflammation in smokers has been proposed as one of the mechanisms involved in initiating and accelerating the atherothrombotic process leading to cardiovascular disease development. Several studies reported that cigarette smoke exposure is associated with increased inflammation. It has been found that the level of serum inflammatory marker such as tumor necrosis factor-α (TNF-α) is significantly higher in smokers compared to non-smokers [2]**.** Quit smoking is the best method to reduce the risk of cardiovascular disease. However, there is still a need to reduce this risk among chronic smokers who fail to quit smoking which in turn leads to an increased interest to investigate the possible beneficial effect of natural products among smokers. Previous study has reported that supplementation of camu-camu juice (with high vitamin C content), which has anti-inflammatory activity, among male smokers for 7 days significantly reduces the levels of serum high sensitivity C-reactive protein (hsCRP), interleukin-6 (IL-6) and interleukin-8 (IL-8) [3].

Honey is a natural product of bees and contains carbohydrates such as glucose and fructose as well as proteins, minerals, organic acids and aromatic compounds [4]. Apart from that, it also contains enzymes such as glucose oxidase and catalase [5], vitamins A and E [6], flavonoids [7, 8], phenolic acids [9], as well as has antioxidant properties [10]. Honey has been demonstrated to possess a potent anti-inflammatory property. It is suggested that protection of murine keratinocytes from ultraviolet radiation-induced inflammation by honey added to cell cultures occurs through the suppression of inflammatory cytokines, ultraviolet B-induced cyclooxygenase-2 expression, inducible nitric oxide synthase protein expression as well as through the production of prostaglandin E_2_ [11]. In animal study, oral treatment with 5 g/kg and 10 g/kg of honey has been shown to reduce the inflammation in an experimentally-induced inflammatory bowel disease in rats [12]. Meanwhile, in human study, honey supplementation at a dose of 70 g daily for 8 weeks significantly reduces seminal plasma inflammatory markers such as TNF-α, IL-6 and IL-8 among male road cyclists [13].

However, to date, no study has been reported to determine whether honey supplementation is able to improve inflammation among smokers. Therefore, the aim of this study was to determine the possible role of honey in improving inflammation among chronic smokers.

## Methods

### Subjects and eligibility

This study involved subjects aged between 20 and 50 years old. Subjects from non-smoker group should have no history of smoking and exposure to environmental tobacco smoke. Meanwhile, subjects from smoker group should have smoked at least 10 cigarettes per day for more than 5 years. Exclusion criteria were the presence of severe infection, regular consumption of dietary supplements and/or multivitamins 3 months prior to the study, obese (BMI > 30 kg/m^2^), taking alcohol or had history of cardiovascular disease. Ethical approval was obtained from Human Research Ethics Committee of Universiti Sains Malaysia (USM) (approval code: JEPeM [243.3.(6)]. This study took place in Physiology Laboratory, School of Medical Sciences, USM from September 2012 to August 2014.

### Sample size

Sample size for each group was calculated using Power and Sample Size Calculation Software version 3.0.10. The assumption of Type I error probability was 5% (0.05) while the power of the study was 80% (0.8). The difference in population means between two groups (δ) was 30.2 pg/mL and within group standard deviation (σ) was 40.1 pg/mL based on IL-6 level from the previous study [13]. The ratio of control to experimental group (m) was standardized at 1. Considering a drop-out rate of 20%, 36 subjects were recruited for each smoker group of the study. However, the final number of subjects for each group was 32 after excluding the dropped out subjects.

### Study design and intervention

**Fig. 1:**
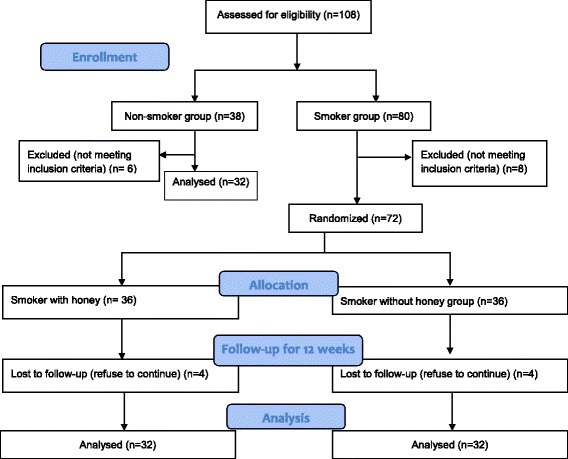
CONSORT Flow diagram of subject recruitment

In this an open-label randomized controlled trial, a total of 108 subjects from USM staff in the Health Campus and Quit Smoking Clinic, USM Hospital were screened for inclusion in the study. Thirty eight from them were screened for non-smoker group but 6 subjects were excluded because of not meeting the inclusion criteria. Meanwhile, 80 subjects were screened for smoker group and 8 subjects were excluded because of not meeting the inclusion criteria. The objectives of the study were carefully explained to the subjects and informed consent was taken. Blood was obtained from all subjects (non-smokers and smokers) during the initial visit (week 0) for the pre-intervention status assessment. Smokers from smoker group were then assigned into 2 parallel groups namely smokers with honey (*n* = 36) and smokers without honey (*n* = 36). Simple randomization was done by the first author using a randomized table created by computer software (Random allocation software version 1.0). The allocation was not concealed and allocation ratio was 1:1. Supplementation of honey (20 g/day orally for 12 weeks) was given to the smokers with honey group at weeks 0, 4 and 8. Honey used in this study was a pure local honey named Tualang honey supplied by Federal Agricultural & Agro Based Industry, Malaysia. The subjects were instructed to return the empty sachet to ensure compliance and any possible side effects of supplementation were monitored. During the study, 4 subjects refused to continue the intervention and the final number of subjects was 32 in smokers with honey group (*n* = 32). Similarly, 4 subjects refused to continue the intervention and the final number of subjects was 32 in smokers without honey group (*n* = 32). Post-intervention status was reassessed after 12 weeks. As for the subject from non-smoker group, blood was obtained only at pre-intervention for baseline comparison on the status of inflammatory markers between non-smoker and smoker groups. All subjects were asked to maintain their current activity levels, which include diet and exercise, during the study. The flowchart of this study is shown in Fig. 1.

### Blood collection and sample preparation

A total of 3 mL of venous blood was obtained from each subject at pre-intervention and from each subject of both smoker groups at post-intervention. The blood was collected into a tube containing ethylenediamine tetraacetic acid. The whole blood in the tube was centrifuged at 1000 x g for 10 min at 4 °C. A hundred μL of plasma was alliquoted into each microcentrifuge tube and kept frozen at −80 °C for biochemical analysis of plasma inflammatory markers which include TNF-α, IL-6 and hsCRP.

### Determination of inflammatory markers

Plasma TNF-α, IL-6 and hsCRP were determined using Human TNF-alpha ELISA Kit (Raybiotech, USA), Human Interleukin 6 ELISA Kit (AssayPro, USA) and Human hs-CRP ELISA Kit (Cusabio, USA), respectively.

### Statistical analysis

Results were analysed using Statistical Package for the Social Sciences version 20. Independent *t*-test was used to analyse the difference of pre-intervention inflammatory markers between non-smoker and smoker groups. The differences between pre and post-intervention inflammatory markers in each group of smokers were assessed by paired *t*-test. A value of *p* < 0.05 was considered statistically significant. Data are presented as mean and standard error of mean (SEM).

## Results

Table 1 shows the baseline characteristics of the subjects. There were no significant differences for mean age and mean arterial pressure (MAP) between non-smoker and smoker groups.

**Table 1:** Characteristics of the subjects between non-smokers and smokers groups

	Non-smokers (*n* = 32)	Smokers (*n* = 64)
Age (years)	35.16 (1.50)	38.38 (0.97)
MAP (mmHg)	89.77 (0.99)	92.57 (1.06)

Data are presented as mean (SEM). MAP: Mean arterial pressure. No significant differences for mean age and mean arterial pressure (MAP) between non-smokers and smokers groups (Independent *t*-test)

### Pre-intervention level and changes (between pre and post-intervention) of inflammatory markers among subjects

The levels of inflammatory markers at pre-intervention are presented in Table 2. The levels of mean plasma TNF-α and mean plasma IL-6 at pre-intervention did not show any significant differences between smoker and non-smoker groups. In contrast, the level of mean plasma hsCRP at pre-intervention was significantly higher in smoker group compared to non-smoker group.

**Table 2:** Pre-intervention inflammatory markers between non-smokers and smokers groups

	Non-smokers (*n* = 32)	Smokers (*n* = 64)
TNF-α (pg/mL)	2755.42 (172.00)	2821.25 (99.71)
IL-6 (ng/mL)	2.37 (0.30)	1.77 (0.16)
hsCRP (ng/mL)	1.58 (0.25)	2.45 (0.24)*

Data are presented as mean (SEM). TNF-α: tumor necrosis factor-alpha; *IL* interleukin; *hsCRP* high sensitivity C-reactive protein. *p < 0.05 compared to non-smokers (Independent *t*-test)

The levels of inflammatory markers at post-intervention are presented in Table 3. At post-intervention, the mean plasma TNF-α was significantly increased while the mean plasma hsCRP was significantly reduced after 12 weeks in smokers with honey group. No significant differences were observed for the levels of mean plasma TNF-α and mean plasma hsCRP in smokers without honey group. Furthermore, the level of mean plasma IL-6 revealed no significant difference in both smokers with honey and smokers without honey groups.

**Table 3:** Pre-intervention and post-intervention inflammatory markers in smokers

	Smokers with honey (*n* = 32)		Smokers without honey (*n* = 32)	
	Pre-intervention	Post-intervention	Pre-intervention	Post-intervention
TNF-α (pg/mL)	2615.86 (145.31)	3855.92 (217.83)*	3026.64 (128.74)	2893.59 (68.36)
IL-6 (ng/mL)	1.60 (0.19)	1.77(0.17)	1.96 (0.26)	1.73 (0.26)
hsCRP (ng/mL)	2.31 (0.32)	1.61 (0.26)*	2.91 (2.38)	1.65 (2.43)

Data are presented as mean (SEM). TNF-α: tumor necrosis factor-alpha; IL: interleukin; hsCRP: high sensitivity C-reactive protein

*p < 0.05 compared to the corresponding pre-intervention (Paired *t*-test)

## Discussion

Inflammation is fundamental to the body’s defence against infection. Furthermore, it is also a critical component of normal tissue repair. Cigarette smoking has been shown to alter the host response and therefore, modifies the progression and outcome of inflammation. The exact mechanisms of cigarette smoking leading to inflammation are not clearly understood. However, it has been postulated that smoking affects a number of inflammatory process through its effect on immune-inflammatory cells, causing an immunosuppressant state and cytokine secretion [14]. Therefore, in this study, the levels of inflammatory markers such as TNF-α, IL-6 and hsCRP were measured in plasma of smokers with and without honey supplementation. Inflammatory markers were also measured at pre-intervention among non-smokers for baseline comparison.

The level of TNF-α at pre-intervention in smoker group was not statistically significant compared to non-smoker group which might suggest that macrophages are not activated to produce TNF-α among smokers in the present study. However, the level of plasma TNF-α revealed a significant increase after 12 weeks of honey supplementation. This result is in accordance with previous in vitro study which shows that 5.8 kDA protein component of honey is able to stimulate the production of TNF-α by macrophages via toll-like receptor (TLR)-4. TLR-4 is able to detect lipopolysaccharides from gram-negative bacteria, thus, plays a role in pathogen recognition and activation of innate immunity. In human monocytes, blocking of the TLR-4 but not TLR-2 receptor significantly inhibits honey-stimulated TNF-α production, thus, suggesting TLR-4 receptor involvement [15]. A family of TLRs serves as primary sensors that recognize a variety of microbial components as well as induce innate immune responses. All pathways of TLR signaling terminate in NF-kB (nuclear factor kappa-light-chain-enhancer of activated B cells activation), a protein complex that controls the inflammatory cytokine genes expression [16]. Therefore, activation of NF-kB may lead to an increase in expression of inflammatory cytokines which include TNF-α, IL-6, IL-8, IL-12 and IL-18 [14]. In the present study, it is therefore plausible to suggest that honey may contribute in activation of NF-kB of macrophages via TLRs leading to increased expression and production of TNF-α. However, the finding on the increased TNF-α level seems to suggest that honey may exert an inflammatory effect, but it needs further study by also measuring other inflammatory markers such as IL-1, IL-4 and IL-10.

The level of plasma IL-6 in the present study was slightly lower in smoker group compared to non-smoker group at pre-intervention but not statistically significant. On the contrary, in other study**,** it is found that there is a significantly higher level of serum IL-6 among smokers compared to non-smokers. The increased level of serum IL-6 indicates a higher level of cytokine-mediated inflammation among elderly active smokers (mean age of 77 years) [17]. These conflicting results may be due to the younger age group (mean age of 38 years) of the subjects involved in the present study or due to the relatively small sample size of the present study. Following 12 weeks of honey supplementation, the level of plasma IL-6 was found to be slightly higher but not statistically significant. However, previous study has shown that honey stimulates IL-6 secretion from human monocytes, leading to an activation of immune response in the cell [18]. Furthermore, honey supplementation at dose of 0.27 ml/kg/orally for 7 days significantly increases the level of mRNA IL-6 expression in *Salmonella typhi* induced of mice [19]. Apart from that, in mice infected with invasive aspergillosis, 10 days honey supplementation at a dose of 1.5 g/kg/orally significantly increases the production of IL-6 as well as improves the work of macrophages perform phagocytosis suggesting that honey can boost the immune system [20]. In contrast, supplementation of honey at a dose of 70 g for 8 weeks to road cyclists significantly shows less elevation in seminal plasma IL-6 suggesting the beneficial effect of honey in reducing seminal plasma IL-6 [13]. In animal study, honey has also been shown to decrease the production of plasma IL-6 and is able to significantly suppress gene and protein expression of IL-6 in paw tissue of inflammatory-induced rat [21]. The inconsistency in these studies compared to the findings of the present study could be explained by the different type and dose of honey administration as well as could be due to the difference response between animal and human following honey supplementation.

In the present study, the level of plasma hsCRP at pre-intervention was significantly higher in smokers compared to non-smokers. This finding may suggest that cigarette smoke may induce vascular inflammatory response leading to atherothrombosis [22]. Following 12 weeks of honey supplementation, the result revealed a significant reduction in plasma level of hsCRP suggesting the anti-inflammatory effect of Tualang honey. Supplementation of antioxidant α-tocopherol, which is present in honey, significantly reduces the levels of CRP in diabetic and non-diabetic patients [23, 24], thus may provide a protective effect against cardiovascular morbidity and mortality. In animal study, honey supplementation for 21 days at a dose of 1.0 g/kg body weight significantly decreases CRP level in diabetic and hypercholesterol rats [25]. Furthermore, honey supplementation for 30 days significantly reduces the serum hsCRP among subjects with elevated hsCRP suggesting that supplementation of honey may reduce inflammation [26]. This is supported by previous study that phenolic compounds present in honey are responsible for the anti-inflammatory activity [27]. The suggested mechanism of action includes the suppression of proinflammatory activities of cyclooxygenase-2 and/or inducible nitric oxide synthase via these flavonoids [28]. Tualang honey has been reported to have flavonoids such as benzoic, gallic, syringic and trans-cinnamic acids and phenolic acids such as catechin and kaempferol [8] which may be responsible for the anti-inflammatory effect of honey as shown by the reduced hsCRP level among smokers and needs further study to evaluate its exact mechanism of action. However, the validity of this biomarker remains unclear as treatment with modified CRP and native CRP have been shown to give opposite effects on atherosclerosis in ApoE(−/−) mice [29].

## Conclusions

In conclusion, chronic smokers had significantly higher levels of hsCRP compared to non-smokers at pre-intervention. Supplementation of honey for 12 weeks significantly increased the level of TNF-α but significantly reduced the level of hsCRP among chronic smokers. These conflicting findings on both these inflammatory markers may suggest that the effect of Tualang honey supplementation on inflammatory process among chronic smoker is inconclusive. Additional assessment on other inflammatory markers such as IL-1, IL-4 and IL-10 may be useful to give a better picture on the complex interplay between inflammatory and anti-inflamatory processes with larger number of subjects in future study.

## Abbreviations

CRP: C-reactive protein
hsCRP: High sensitivity C-reactive protein
IL-6: Interleukin-6
IL-8: Interleukin-8
MAP: Mean arterial pressure
NF-kB: Nuclear factor kappa-light-chain-enhancer of activated B cells activation.
SEM: Standard error of mean
TLR: Toll-like receptor
TNF-α: Tumor necrosis factor-α
USM: Universiti Sains Malaysia
